# Breast density measurement for personalised screening

**DOI:** 10.1186/bcr3272

**Published:** 2012-11-09

**Authors:** JC Sergeant, S Musa, M Wilson, DG Evans, A Howell, SM Astley

**Affiliations:** 1 Institute of Population Health, University of Manchester, UK; 2 Manchester Medical School, University of Manchester, UK; 3 Nightingale Centre and Genesis Prevention Centre, University Hospital of South Manchester, Manchester, UK

## Introduction

Breast density is both a modifiable risk factor for breast cancer and an indicator of the sensitivity of mammography. Reliable measurement in the screening population could enable personalised screening to maximise early detection of cancer. In the Predicting Risk of Cancer at Screening (PROCAS) study, we are investigating different approaches to measuring breast density, and we present comparative data from one subjective method and two automated volumetric methods.

## Methods

The screening mammograms of 4,109 women enrolled in PROCAS were visually assessed independently by two experienced film readers, who recorded their estimates of percentage density on visual analogue scales (VAS). The mammograms were also processed by Quantra™ (Hologic Inc.) and Volpara (Matakina Technology Ltd). They were ranked according to density by each method, and the top 10% and 1% compared.

## Results

Of the 617 mammograms ranked in the most dense 10% by at least one method, only 127 were high density by all three methods. The overlap between the two volumetric methods was 214; between VAS and Quantra™ it was 195 and between VAS and Volpara it was 147. For the 48 mammograms in the top 1% by density, the overlaps were 13, 9 and 7, respectively.

## Conclusion

The lack of overlap between the methods was surprising and has serious implications for the implementation of personalised screening. The optimum measure of breast density in digital mammograms has yet to be identified; different methods may yield the strongest links with cancer risk and sensitivity of mammography, and further research is needed to elucidate these relationships.

